# 7.3 EVALUATING THE NEUROBIOLOGICAL CORRELATES AND IMPACT OF TREATMENT ON COGNITIVE DYSFUNCTION IN ADHD AND SCHIZOPHRENIA BY MEANS OF THE PATTERN ELECTRORETINOGRAM

**DOI:** 10.1093/schbul/sby014.025

**Published:** 2018-04-01

**Authors:** Emanuel Bubl, Lisa Werner, Yumin Liang, Dieter Ebert, Evelyn Friedel, Anna Bubl, Michael Bach, Ludger Tebartz van Elst

**Affiliations:** 1 University of Saarland; 2 University of Freiburg

## Abstract

**Background:**

Problems with cognitive function are found in many major psychiatric disorders. In schizophrenia and attention deficit hyperactivity disorder (ADHD), they are among the core symptoms. Based on a growing recognition that there is little diagnostic specificity for any single cognitive impairment, there is an increasing emphasis on investigating impairments across psychiatric disorders. This approach, which is consistent with the NIMH Research Domain Criteria initiative, is expected to lead to a better understanding of the neurobiological mechanisms involved in cognitive deficits.

An increase in neuronal background noise has been identified as a neuronal correlate of inattention. Because the dopamine system has been found to play a critical role in modulating neuronal noise, dopamine dysfunction may play a substantial role in generating the excessive noise that has been found to characterize information processing in both schizophrenia and ADHD. This issue can be studied noninvasively via electrophysiological examination of the retina, a distinct neural network. Both basic research and human studies indicate that retinal information processing is under strong dopaminergic modulation (Bubl, Biol. Psychiatry, 2010; Bubl, Br J Psychiatry, 2012). We have previously demonstrated an elevated level of background noise at a very early stage in visual information processing in untreated patients with ADHD (Bubl, Plos One, 2015). Moreover, background noise was associated with inattention measures in these subjects. To further address the hypothesis that elevated retinal noise reflects dopaminergic dysfunction, we report here on a new study that compared retinal background noise in patients with ADHD both before and after therapy, as well as in patients with schizophrenia.

**Methods:**

Neuronal noise was assessed using pattern electroretinogram (PERG), an objective electrophysiological measure for retinal network function from the photoreceptors to the retinal ganglion cells. A total of 20 patients diagnosed with ADHD were tested both before and after treatment with methylphenidate (MPH). The control group consisted of 21 healthy subjects. The PERGs were recorded in a steady state mode in response to checkerboard stimuli of 12 reversals/s. Data collection with people with schizophrenia is ongoing, and results will be reported at SIRS.

**Results:**

Before treatment, the patients with ADHD presented with elevated background noise (higher by 127%) in comparison to the control group. After treatment, noise level did not differ from what was observed in the control group. Retinal background noise was found to be highly correlated with the severity of the ADHD symptoms. The results will be discussed in relationship to our findings in patients with schizophrenia.

**Discussion:**

These data provide further evidence for the hypothesis that elevated background noise is linked to ADHD and cognitive deficits. The findings are of special relevance because ADHD is a disorder with a dedicated treatment option for cognitive symptoms. Interestingly, a similar pathophysiological mechanism for cognitive dysfunction has been proposed for both schizophrenia and ADHD. However, because ADHD medications, such as MPH, typically elevate dopamine levels, potentially leading to exacerbation of psychotic symptoms, different approaches for treating cognitive symptoms in schizophrenia need to be explored. On this basis, current approaches used to target neuronal noise and cognitive symptoms in patients with schizophrenia will be discussed and their relevance for future research will be addressed.

